# Supplementary material for: Reconciling Mining with the Conservation of Cave Biodiversity: A Quantitative Baseline to Help Establish Conservation Priorities
Source: PLoS One. 2016 Dec 20;11(12):e0168348. doi: 10.1371/journal.pone.0168348 (PMC5173368; doi:10.1371/journal.pone.0168348)
Supplement: S1 Dataset — (ZIP) [file pone.0168348.s002.zip › Taxa/Serra Norte/SN_2013/N5S/Lista N5S-10.pdf]

## CAVIDADE N5S-0010

| Classe | Ordem | Fam/Outros | Gên/Outros | Espécie | S-U | U-S | Zon. |
|---|---|---|---|---|---|---|---|
| Annelida | Oligochaeta | | | jovem | 2 | | A |
| Arachnida | Acari | Cunaxidae | | sp.1 | X | | A |
| Arachnida | Acari | Eupodidae | | sp.1 | X | | A |
| Arachnida | Acari | Ixodidae | | jovem | X | | P/A |
| Arachnida | Acari | Mesostigmata | | jovem | X | | E/A |
| Arachnida | Acari | Rhagidiidae | | sp.20 | X | | E |
| Arachnida | Acari | Trombidiformes | | sp.49 | X | | A |
| Arachnida | Acari | | | jovem | | X | |
| Arachnida | Amblypygi | Charinidae | *Charinus* | sp.1 | 4 | | P/A |
| Arachnida | Amblypygi | Phrynidae | *Heterophrynus* | *longicornis* | 3 | X | |
| Arachnida | Araneae | Araneidae | *Parawixia* | *kochi* | X | | A |
| Arachnida | Araneae | Barychelidae | | jovem | 1 | | E |
| Arachnida | Araneae | Caponiidae | | sp.1 | X | X | E/P/A |
| Arachnida | Araneae | Ctenidae | | jovem | | 1 | |
| Arachnida | Araneae | Drymusidae | *Drymusa* | *spelunca* | X | | E/P/A |
| Arachnida | Araneae | Filistatidae | | jovem | X | | E |
| Arachnida | Araneae | Mysmenidae | *Microdipoena* | sp.1 | X | | E |
| Arachnida | Araneae | Ochyroceratidae | *Ochyrocera* | sp.1 | X | | E/P/A |
| Arachnida | Araneae | Ochyroceratidae | *Speocera* | sp.1 | X | | A |
| Arachnida | Araneae | Oonopidae | aff. *Xyccarph* | sp.3 | X | | E |
| Arachnida | Araneae | Pholcidae | *Mesabolivar* | sp.1 | X | | P |
| Arachnida | Araneae | Pholcidae | Ninetinae | sp.1 | X | | E |
| Arachnida | Araneae | Prodidomidae | *Lygromma* | sp.2 | X | | E |
| Arachnida | Araneae | Salticidae | | sp.6 | X | | E |
| Arachnida | Araneae | Scytodidae | *Scytodes* | jovem | 1 | | E |
| Arachnida | Araneae | Theridiidae | *Theridion* | sp.1 | X | | P/A |
| Arachnida | Araneae | Theridiosomatidae | *Plato* | sp.1 | X | X | E/P/A |
| Arachnida | Opiliones | Escadabiidae | | sp.1 | X | X | A |
| Arachnida | Opiliones | Escadabiidae | | sp.2 | X | X | E/A |
| Arachnida | Opiliones | Manaosbiidae | *Saramacia* | sp.1 | 1 | | E |
| Arachnida | Pseudoscorpiones | Chthoniidae | *Pseudochthonius* | sp.1 | X | | E/P |
| Chilopoda | Geophilomorpha | Schendylidae | | sp.1 | 2 | | A |
| Chilopoda | Scutigeromorpha | Pselliodidae | *Sphendononema* | *guildingii* | 7 | X | E/P/A |
| Diplopoda | Polydesmida | Pyrgodesmidae | | sp.1 | X | X | A |
| Diplopoda | Spirostreptida | Pseudonannolenidae | *Pseudonannolene* | jovem | 1 | | E |
| Entognatha | Collembola | Cyphoderidae | | sp.1 | X | | E |
| Entognatha | Collembola | Isotomidae | | sp.1 | X | | E |
| Entognatha | Collembola | Paronellidae | | sp.1 | X | | A |
| Entognatha | Collembola | Paronellidae | | sp.4 | X | | E |
| Entognatha | Collembola | Sminthuroidea | | sp.2 | X | X | P/A |
| Entognatha | Diplura | Campodeidae | | sp.1 | X | | E |
| Insecta | Blattodea | | | jovem | 1 | X | E |
| Insecta | Coleoptera | Carabidae | | sp.3 | X | | E/A |
| Insecta | Coleoptera | Curculionidae | | sp.1 | X | | A |
| Insecta | Coleoptera | Dytiscidae | | sp. | | X | |
| Insecta | Coleoptera | Dytiscidae | | sp.5 | X | | P |
| Insecta | Coleoptera | Dytiscidae | | sp.12B | X | | P |
| Insecta | Coleoptera | Dytiscidae | | sp.13 | X | | P |
| Insecta | Coleoptera | Scydmaenidae | | sp.3 | X | | A |

| | | | | | | | |
|---|---|---|---|---|---|---|---|
| Insecta | Coleoptera | Staphylinidae | Pselaphinae | sp.12 | X | | A |
| Insecta | Diptera | Culicidae | *Culex (Aedinus)* | sp. | | X | |
| Insecta | Diptera | Psychodidae | Phlebotominae | sp. | X | | E/P/A |
| Insecta | Diptera | Psychodidae | *Pifanomyia* | *gruta* | | X | |
| Insecta | Diptera | Psychodidae | *Sciopemyia* | *sordellii* | | X | |
| Insecta | Diptera | Tachinidae | | sp. | X | | P |
| Insecta | Heteroptera | Dipsocoroidea | | jovem | X | | P |
| Insecta | Heteroptera | Reduviidae | Triatominae | sp. | 3 | | E |
| Insecta | Heteroptera | Veliidae | *Paravelia* | sp. | X | X | E/P/A |
| Insecta | Homoptera | Auchenorrhyncha | | jovem | | X | |
| Insecta | Homoptera | Cixiidae | | jovem | X | X | E/P |
| Insecta | Hymenoptera | Diapriidae | | sp.1 | X | | A |
| Insecta | Hymenoptera | Formicidae | *Camponotus* | sp.1 | X | | E/P |
| Insecta | Hymenoptera | Formicidae | *Camponotus* | sp.2 | X | | E/P |
| Insecta | Hymenoptera | Formicidae | *Dolichoderus* | aff. *bispinosus* | X | | E |
| Insecta | Hymenoptera | Formicidae | *Gnamptogenys* | sp.1 | X | X | A |
| Insecta | Hymenoptera | Formicidae | *Hypoponera* | sp.1 | X | X | E |
| Insecta | Hymenoptera | Formicidae | *Pachycondyla* | sp.2 | X | X | E/P/A |
| Insecta | Hymenoptera | Formicidae | *Pheidole* | sp.4 | X | | E |
| Insecta | Hymenoptera | Formicidae | *Trachymyrmex* | sp.1 | X | | E/P |
| Insecta | Isoptera | | | sp. | X | | P |
| Insecta | Lepidoptera | Tineoidea | | sp.1 | X | X | E |
| Insecta | Orthoptera | Phalangopsidae | *Paraclodes* | sp. | | X | |
| Insecta | Orthoptera | Phalangopsidae | *Phalangopsis* | sp. | 35 | | E |
| Insecta | Psocoptera | Epipsocidae | *Epipsocus* | sp.2 | X | | E |
| Insecta | Psocoptera | Epipsocidae | *Mesepipsocus* | sp.1 | X | | E |
| Insecta | Psocoptera | Psyllipsocidae | | jovem | X | | E |
| Insecta | Thysanura | Nicoletiidae | Nicoletiinae | sp.1 | | X | |
| Gastropoda | Pulmonata | Subulinidae | | sp.5 | X | | A |
| Gastropoda | Pulmonata | Systrophiidae | *Happia* | sp. | X | X | E/P/A |
| Malacostraca | Decapoda | | | sp. | | 1 | |
| Malacostraca | Isopoda | Armadillidae | | sp.1 | X | | E |
| Malacostraca | Isopoda | Scleropactidae | *Circoniscus* | *buckupi* | X | | A |
| Malacostraca | Isopoda | Scleropactidae | | sp. | | X | |
| Onychophora | Onychophora | Peripatidae | *Peripatus* | sp.1 | 1 | | E |
| Amphibia | Anura | Strabomantidae | *Pristimantis* | cf. *fenestratus* | 4 | | E/P |
| Mammalia | Chiroptera | Emballonuridae | *Peropteryx* | sp. | 20 | | P/A |
| Mammalia | Chiroptera | Furipteridae | *Furipterus* | *horrens* | 5 | | |
| Mammalia | Chiroptera | Phyllostomidae | *Diphylla* | *ecaudata* | | X | P/A |
| Mammalia | Rodentia | Cricetidae | *Rhipidomys* | sp. | | 1 | |
